# eHealth System for Collecting and Utilizing Patient Reported Outcome Measures for Personalized Treatment and Care (PROMPT-Care) Among Cancer Patients: Mixed Methods Approach to Evaluate Feasibility and Acceptability

**DOI:** 10.2196/jmir.8360

**Published:** 2017-10-02

**Authors:** Afaf Girgis, Ivana Durcinoska, Janelle V Levesque, Martha Gerges, Tiffany Sandell, Anthony Arnold, Geoff P Delaney, The PROMPT-Care Program Group

**Affiliations:** ^1^ Centre for Oncology Education and Research Translation Ingham Institute for Applied Medical Research Liverpool Australia; ^2^ South Western Sydney Clinical School Faculty of Medicine The University of New South Wales Liverpool Australia; ^3^ Illawarra Cancer Care Centre Wollongong Hospital Wollongong Australia; ^4^ Liverpool Cancer Therapy Centre Liverpool Hospital Liverpool Australia

**Keywords:** patient reported outcome measures, eHealth, self-management, medical oncology, patient-centered care, electronic health records

## Abstract

**Background:**

Despite accumulating evidence indicating that collecting patient-reported outcomes (PROs) and transferring results to the treating health professional in real time has the potential to improve patient well-being and cancer outcomes, this practice is not widespread.

**Objective:**

The aim of this study was to test the feasibility and acceptability of PROMPT-Care (Patient Reported Outcome Measures for Personalized Treatment and Care), a newly developed electronic health (eHealth) system that facilitates PRO data capture from cancer patients, data linkage and retrieval to support clinical decisions and patient self-management, and data retrieval to support ongoing evaluation and innovative research.

**Methods:**

We developed an eHealth system in consultation with content-specific expert advisory groups and tested it with patients receiving treatment or follow-up care in two hospitals in New South Wales, Australia, over a 3-month period. Participants were recruited in clinic and completed self-report Web-based assessments either just before their upcoming clinical consultation or every 4 weeks if in follow-up care. A mixed methods approach was used to evaluate feasibility and acceptability of PROMPT-Care; data collected throughout the study informed the accuracy and completeness of data transfer procedures, and extent of missing data was determined from participants’ assessments. Patients participated in cognitive interviews while completing their first assessment and completed evaluation surveys and interviews at study-end to assess system acceptability and usefulness of patient self-management resources, and oncology staff were interviewed at study-end to determine the acceptability and perceived usefulness of real-time PRO reporting.

**Results:**

A total of 42 patients consented to the study; 7 patients were withdrawn before starting the intervention primarily because of changes in eligibility. Overall, 35 patients (13 on treatment and 22 in follow-up) completed 67 assessments during the study period. Mean completeness of patient-reported data was 93%, with 100% accuracy of data transfer. Ten patients completed cognitive interviews, 28 completed evaluation surveys, and 14 completed evaluation interviews at study-end. PROMPT-Care patient acceptability was high—100% (28/28) reported the time to complete the Web-based assessments (average 15 min) as *about right*, most willing to answer more questions (79%, 22/28 yes), 96% (27/28) found the Web-based assessment *easier or same as* completing a paper copy, and they valued the self-management resources *.* Oncology staff (n=5) also reported high acceptability and potential feasibility of the system.

**Conclusions:**

Patients and oncology staff found the PROMPT-Care system to be highly acceptable, and the results suggest that it would be feasible to implement it into an oncology setting. Suggested modifications to the patient assessment survey, clinician access to the reports, and system requirements will be made as part of the next stage of large-scale testing and future implementation of the system as part of routine care.

**Trial registration:**

Australian New Zealand Clinical Trials Registry ACTRN1261500135294; https://www.anzctr.org.au/Trial/Registration/TrialReview.aspx?id=369299&isReview=true (Archived by WebCite at http://www.webcitation.org/6lzylG5A0).

## Introduction

Routinely collecting and utilizing patient-reported outcome (PRO) measures enables better patient-centered care [1-4]. Recently published research demonstrates improvements in clinical and health service outcomes, including reduced emergency room visits, longer tolerability of chemotherapy, and improved short- and long-term survival [5,6].

Our published protocol [7] details the proposed methodology for developing and testing the acceptability and feasibility of PROMPT-Care (Patient Reported Outcome Measures for Personalized Treatment and Care), an electronic health (eHealth) system that supports routine collection and analysis of cancer patients’ PROs, real-time feedback of PRO results to their cancer care team to inform patient-centered care, and delivery of evidence-based self-management information to address patient-reported problems. As reported in the protocol, the key feature distinguishing PROMPT-Care from previous oncology-based eHealth systems is its integration into the hospital’s point-of-care oncology information system (OIS).

The objectives of our feasibility study were as follows:

1. To develop an eHealth system that is integrated into the OIS (MOSAIQ, Elekta) to support the assessment of cancer patients’ PROs through the use of electronically administered standardized assessment tools, provision of *real-time* feedback of the results to their treating clinicians, and generation of links to self-management resources for patients, which are tailored to their PROs. This includes developing a production version of the PROsaiq prototype system [8].
2. To implement the pilot version of PROMPT-Care at two hospitals and test the feasibility and functionality of the system.
3. To test the acceptability of the pilot version of PROMPT-Care in a sample of cancer patients and clinicians at the two participating hospitals.

The term *patient* used throughout this document encompasses all people diagnosed with cancer who are currently on treatment and in follow-up.

## Methods

The detailed study methods have been previously outlined in the study protocol [7]. The following is a summary of these methods.

### Study Design and Objectives

#### Setting

The feasibility study was undertaken in the cancer centers of two public hospitals, Liverpool and Wollongong, in New South Wales, Australia. Ethics approval was obtained from the Human Research Ethics Committee of South Western Sydney Local Health District (Reference Number HREC/14/LPOOL/405), with Site Specific ethics approvals obtained for Liverpool Hospital and Wollongong Hospital.

#### Development of the PROMPT-Care eHealth System

To facilitate the development of key clinical and technical aspects of the PROMPT-Care system, a clinical advisory group and a technical advisory group were established, as detailed in the protocol [7].

##### Selection of PRO Measures and Assessment Frequency

The clinical advisory group identified distress, symptoms, and unmet needs as the PRO domains to include in this initial feasibility study, with these domains being the most important for informing patient care and most amenable to evidence-based intervention. Following a comprehensive review of measures, the Distress Thermometer [9] with the problem checklist [10], the Edmonton Symptom Assessment Scale (ESAS) [11], and the Supportive Care Needs Survey-Screening Tool 9 (SCNS-ST9) [12] were selected for inclusion in the PROMPT-Care assessment. For each of the selected PRO measures, item or scale cut-off scores differentiating between *normal* (below threshold—no intervention required) and *clinical* (above threshold and therefore flagged for review or intervention) responses were determined. For each measure, *clinical* thresholds were as follows: a score ≥5 for DT [9], any item checked *yes* for the Distress Thermometer checklist items, a score of ≥4 for ESAS items [11], and a rating of 4 or 5 (ie, moderate or high unmet need) on the SCNS-ST9 [12].

The clinical advisory group also determined the frequency of patients completing the PRO assessments as approximately every 2 weeks for patients who were currently receiving treatment and approximately monthly for those who were in follow-up. It was agreed that the feasibility and acceptability testing would inform future assessment frequency.

##### Development of Algorithms to Guide Response to PROs

A multidisciplinary clinical algorithms working group (n=8; medical and radiation oncologists, social worker, clinical psychologist, and care coordinators) developed actionable recommendations for each item that breaches the clinical threshold, with a total of 15 recommendations developed across the main categories of (1) *No action required*, (2) *Consider reasons for concern and, if required, refer to (types of specialties indicated here, depending on issue) for further assessment and care*, (3) *Clinically address as appropriate OR refer to (types of specialties indicated here, depending on issue) for further assessment and care*, and (4) *Address (type of) needs and identify appropriate sources of support and information.*

##### Development of PRO Clinical Feedback Reports

Two report formats were developed in consultation with the clinical advisory group members: (1) a summary report of the patient’s most recent PROMPT-Care assessment, which included the relevant actionable recommendations (from the 15 developed by the clinical algorithms working group) and (2) a longitudinal report summarizing the PROs over time. The reports utilized graphics and colors to readily highlight issues of patient concern—samples of these reports are included in the protocol publication [7].

##### Collation and Review of Patient Self-Management Resources

A self-management working group (subgroup of the clinical advisory group) identified and reviewed readily available self-management resources in each of the PROMPT-Care assessment domains with those meeting the selection criteria [7] then included on the five domain-specific pages (physical well-being, emotional well-being, social and family well-being, practical support, and maintaining health and well-being) hosted on the Cancer Institute NSW (CINSW) eviQ website [13]. Patients received links only to the pages that were relevant to them, that is, where their scores on any item in that domain breached threshold. Additionally, all resource pages included national cancer support services such as the Cancer Council and Lifeline hotlines and the emergency services.

#### Participants

##### Patients

At the two participating sites, patients who were currently receiving cancer care (including follow-up care) or had recently been diagnosed with cancer and were scheduled to commence cancer treatment were eligible to participate. Eligibility criteria included (1) having a confirmed diagnosis of cancer, (2) aged 18 years or older, (3) cognitively able to provide informed consent and understand the assessments, and (4) having sufficient English skills to complete the survey in English. Exclusion criteria were (1) having a diagnosis of a blood cancer and (2) not having access to the Internet outside the clinic.

##### Staff

All staff who provided care in the oncology departments at the participating hospitals during the study period were eligible to participate, with the exception of those who were directly involved in the development of key aspects of the PROMPT-Care system (GPD and AM).

#### Procedure

The following is a summary of the feasibility study procedures. More detailed procedures are included in the protocol [7].

##### Oncology Team Training

During the setup phase, oncologists and other staff (including nurse care coordinators and allied health staff) from the two participating cancer centers were introduced to the PROMPT-Care program through presentations and training resources to explain the purpose of PROMPT-Care and how to access and interpret the reports.

##### Patient Recruitment

Participating clinicians reviewed their patient lists for the upcoming 4 to 6 weeks and identified eligible patients who were then mailed an information and consent pack and telephoned by the research staff to confirm eligibility and to obtain verbal consent. Participants were then assigned a unique study identifier to ensure anonymity during analysis. Consenting patients attended a PROMPT-Care appointment 20 min before their upcoming scheduled appointment at the cancer center to complete study paperwork and their first PROMPT-Care assessment, with research staff available to assist patients who needed help completing the assessments.

##### PROMPT-Care Assessments

**Figure 1:**
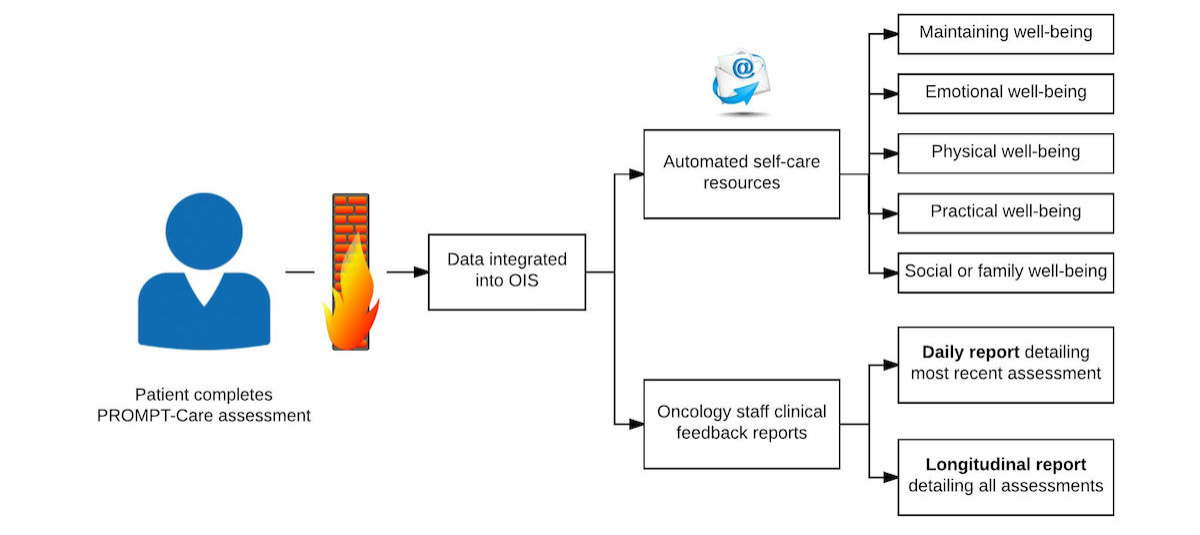
Simplified representation of the PROMPT-Care pilot eHealth system.

Patients who were on treatment completed the PROMPT-Care assessment every 2 to 4 weeks, depending on the schedule of their review appointments, and those on follow-up completed assessments approximately monthly. Patients attending the clinic for an appointment completed the PROMPT-Care assessment in the waiting area using an electronic tablet device provided by the research team, and follow-up patients either completed their PROMPT-Care assessment from home via a link sent by email or in the clinic if they were attending for a review appointment. Patients who were due to complete their PROMPT-Care assessments from home were sent one reminder email if they had not completed it within the requested time frame (48 hours). Submitted data were stored on a secure server hosted by the hospital OIS (MOSAIQ). To ensure successful transfer of assessment data to the OIS, two patient identifiers, surname and unique medical record number, were used at survey log-in. Patients were able to review and change responses by navigating back and forward buttons and were able to save a draft copy before submission. An overview of the PROMPT-Care pilot eHealth system is described in Figure 1.

##### Access and Review of Reports

All patients participating in this PROMPT-Care feasibility study were flagged as *PROMPT-Care Trial* participants on the OIS used by the participating sites, with clinicians instructed to access the report during the consultation, review any issues flagged as problematic by the patient (ie, scores above threshold), discuss these with the patient, and take any appropriate actions to address the issues.

##### Patient Self-Management

Upon completion of the PROMPT-Care assessment, patients received an email with links only to the website pages of the domains in which they breached threshold scores on any of the items in that domain. Patients who scored below threshold on all items received the link to a *maintaining health and well-being* page.

##### Evaluation of Acceptability of PROMPT-Care

The functionality and acceptability of the PROMPT-Care eHealth system was tested at the two participating hospitals with a focus on the assessment of the accuracy and completeness of data transfer procedures (from the point of the patient completing an assessment to a report appearing via MOSAIQ), the extent of missing data from participants’ assessments, the acceptability of the eHealth system and usefulness of the self-management resources, and the acceptability and perceived usefulness of the real-time PRO reporting.

##### Cognitive Interviews

A subset of participants volunteered to take part in cognitive interviews [14], including a combined think-aloud and verbal probing technique [15], the first time they completed the PROMPT-Care assessment. The cognitive interviews were used to identify issues with participant item comprehension, recall, and judgment and ability to use the Web-based PROMPT-Care assessment tool.

##### Patient Surveys and Interviews

Participants completed a Web-based evaluation survey at the end of the trial period. The evaluation survey explored several elements of user acceptability and feasibility such as device usage, attitude toward electronic PRO collection (eg, privacy, ease of completion, and time to complete), willingness to answer more questions, and preferred frequency of assessment completion. They also rated the perceived usefulness of the self-management resources and review of clinical feedback reports during consultations. A subset of participants were invited (based on their evaluation survey responses) to participate in a brief semistructured telephone interview to further explore their experience with the eHealth system and the usefulness of the self-management resources.

##### Oncology Staff Interviews

Participating oncology staff were invited to participate in a brief semistructured telephone interview at study completion to provide feedback on the feasibility and acceptability of the PROMPT-Care system. Specifically, the interviews explored the ease of access, relevance of content, and usefulness of the feedback reports.

##### Data Transfer and Completeness

Patient data regarding clinical details, response counts, PROMPT-Care assessments, and time taken to complete each assessment were extracted from the OIS system and provided to the research team. Additionally, user and technical errors observed were monitored and recorded by research staff.

### Analysis

Descriptive statistical analysis was conducted using OIS system data and patient evaluation surveys. User and technical errors recorded in error logs were analyzed using content analysis. All patient and health care provider interviews were digitally recorded and transcribed verbatim. Evaluation interviews were analyzed using thematic content analysis. Four experienced psycho-oncology or health services researchers (JL, ID, TS, and MG) reviewed the transcripts independently and developed preliminary coding schedules, with discrepancies resolved through discussion and consensus. Cognitive interviews were analyzed using content analysis. Transcripts were reviewed by 2 researchers (TS and HC) using a coding framework adapted from Willis [15], with differences resolved by a third reviewer (ID) and consensus reached.

## Results

### User Characteristics

During the 3-month recruitment period, 205 patients were approached, and 42 (20.5%) patients consented to participate in the program. Seven patients were withdrawn before starting the program: one because of health issues, 2 patients because of changes in personal circumstances, and 4 participants were no longer contactable by email. Overall, 35 patients were involved in testing the usability of the PROMPT-Care system, of whom 28 completed evaluation surveys, 14 participated in evaluation interviews, and 10 participated in cognitive interviews. Table 1 lists participants’ clinical and demographic information. Mean age of participants was 62 years (range: 39-85 years; standard deviation=11.2), and 69% (24/35) of the participants were female. Participants had been diagnosed with a range of cancers; 13 participants were currently receiving active treatment and 22 were receiving follow-up care. Patients reported diverse views on their preferred frequency of assessment completion, with 36% (10/28) preferring specific milestones in treatment (eg, start or end of treatment and first follow-up visit), 33% (9/28) at specific time points (eg, monthly, quarterly, and biannually), and 21% (6/28) preferred completing assessments before every clinic appointment.

The 5 oncology staff involved in the pilot study included 1 medical oncologist, 2 radiation oncologists, 1 nurse, and 1 health services manager.

### Feasibility Evaluation

#### Assessment of Data Completeness

Overall, participants completed 67 (77%, 67/87) assessments, each comprising 67 items (including demographics), totalling 4489 data items. Most (63%, 22/35) participants had completed at least two assessments, with 9 participants completing three assessments and 1 participant completing four assessments. Completeness of PROMPT-Care assessment survey data was high, with only 6.48% (291/4489) missing items. The items that were most commonly skipped by participants were assessment start and completion times and some demographic variables, which together represented 29.2% (85/291) of all missing data. No other pattern of missing data was observed among the remainder of items.

#### Data Transfer

Eight (12%, 8/67) instances of failed survey submissions from the point of a patient completing an assessment to initial data transfer into the OIS were observed. Each problem was investigated and resolved by study staff and assessment data subsequently transferred into the OIS. Errors experienced were due to either technical issues (n=5) such as rejection of data parameters by the OIS and Wi-Fi or browser problems within the clinics or were a result of patient end user errors (n=3) where participants entered incorrect patient identifiers such as surname or personal medical record numbers. However, once the errors were addressed and data were successfully received in the OIS, the accuracy of data transfer from the OIS to presentation in clinical feedback reports was 100.00% (4489/4489) across all data items submitted, with no errors in patient data noted in the reports.

### Acceptability Evaluation

#### Patient Feedback

##### Usability of PROMPT-Care Tool to Complete Assessments

Overall, cognitive interviews (mean time: 30 min and range: 17-42 min) demonstrated that patients completed the PROMPT-Care assessments with ease and indicated that the items were not confronting or upsetting and that they captured all of their concerns. Most participants demonstrated a high understanding of the questions and were able to follow instructions appropriately. Additionally, no patients reported having difficulty changing response options for the different question sets and were easily able to adapt to the scales of the instrument. One participant felt that the variety of response formats (Distress problem checklist, *yes or no*, ESAS numeric *0-10* scale, and SCNS TS-9 5-point Likert scale *no need-* *high need*) should be retained, as a single response type such as yes or no held potential for automated thoughtless assessment completion and could lead to error.

Of the 10 participants who completed a cognitive interview, 2 demonstrated difficulty with recall strategies and appeared to answer items outside the instrument time frames. They were also observed to answer questions generally and not specifically in relation to their cancer experience and/or care.

The Distress Thermometer was presented as a graphic in the Web-based assessment, in line with its original display [16], with patients required to slide a bar up to the score signifying their level of distress (0-10). However, half of the cognitive interview participants experienced difficulty completing this item, particularly with reading the font and selecting their desired response option because of the widget layout. They often required assistance from the interviewer to input their score.

Additionally, minor issues in comprehension and judgment errors were observed among a variety of items, for example, insurance, family health issues, housing, feeling swollen, and fear of cancer spreading. However, they were deemed unproblematic as participants self-resolved queries and provided reasonable insight into their thought process and how they arrived at their responses.

**Table 1:** Participant characteristics (N=35).

Participant characteristic		n (%)
**Age in years**		
	Range	39-85
	Mean (standard deviation)	62.23 (11.2)
**Sex**		
	Male	11 (31)
	Female	24 (69)
**Site of cancer**		
	Breast	20 (57)
	Gastrointestinal	7 (21)
	Gynecological	1 (3)
	Prostate	7 (20)
**Treatment**		
	Surgery	14 (25)
	Chemotherapy	17 (31)
	Radiotherapy	24 (44)
**Patient type**		
	Active treatment	13 (37)
	Follow-up	22 (63)
**Relationship status^a^**		
	Single	7 (20)
	Married or partnered	26 (74)
**Education status^a^**		
	Secondary school	13 (37)
	Postsecondary education	20 (57)
**Employment^a^**		
	Employed	12 (34)
	Retired	19 (54)
	Other	1 (3)

^a^Some level of missing data.

##### Use and Satisfaction With PROMPT-Care Assessments

Overall, patients reported high acceptability and valued completing the assessments, as expressed below:

It actually gave me a handle to express something that I hadn’t—couldn’t figure out how to express to the person [doctor] I was speaking to, and it prompted them to ask me...it allowed me to have a clear avenue of what I wanted to say

PT01, patient interview

I do really think it is an excellent idea and it’s a valuable tool.

PT02, patient interview

It [PROMPT-Care assessment] makes you think about yourself when you’re completing the questionnaires, which is something we tend to overlook sometimes.

PT03, patient interview

Patients (n=28) who actively used PROMPT-Care were surveyed about their acceptance of the eHealth system, satisfaction with the self-care resources, and use of clinical feedback reports during consultations. All patients reported the time to complete assessments (mean time: 15 min) was *about right*, and most were willing to answer more questions (79%, 22/28 yes).

All participants reported they had enough privacy to complete their assessments, and 96% (27/28) had no concerns about which oncology staff member was going to review their responses. The only participant who expressed some concern about staff reviewing their assessments did not have any issues requiring additional support and, as a result, did not realize that his or her clinical feedback report would be reviewed:

Not looking for call to action based on my results. I found that invasive. I assumed you were using data to improve ongoing care and support for general public.

PT04, evaluation survey

Additionally, most patients (75%, 21/28) found electronic patient-reported assessments to be easier than completing assessments by pen-and-paper; 21% (6/28) found it to be similar, whereas only one (4%) participant found it to be more difficult.

##### Satisfaction With Self-Management Resources

Almost half (43%, 12/28) of the patients spent between 11 to 20 min reviewing the self-management resources at any one time, and 39% (11/28) reported they were very satisfied with the resources provided. Whereas most patients felt the self-management resources were easy to understand and navigate (61%, 17/28) and were easy to access from the link sent via email (61%, 17/28), only half (54%, 15/28) reported they were relevant to their concerns (see Table 2).

Furthermore, qualitative interviews showed that most patients valued the self-management resources, noting that they provided relevant information and came from reliable approved sources. Patients felt that the information prompted them to be more engaged in their own health:

I changed my diet quite a lot...all the information encouraged me...you realize you got to do these things if you want to get better.

PT05, patient interview

...I found them [self-management resources] very interesting. Being able to go over to different sites and suggestions...I found them useful.

PT03, patient interview

It’s [self-management resources] probably the ones I have referred to when I was first diagnosed and researching for what was going on, and also amplifies or complements all the booklets. They’re very well-designed and easy to navigate.

PT05, patient interview

It’s pretty good. I’ve been having treatment for 4.5 years now and the self-management resources—I knew quite a bit of it but there were still a few ideas that were new to me. But I think as a new patient, they’re excellent.

PT06, patient interview

However, some patients expressed a desire for more targeted resources specific to their needs and suggested that the level of tailoring needs to be improved. They considered the advice listed on the pages as general, with resources addressing a variety of issues on the Web page. All self-management resource links relating to each corresponding domain of well-being were presented on a single page, whereas patients indicated they would have preferred an email with resources only related to the specific items they had issues with. The participants stated:

The email links for the self-management resources were just a link to the main website and should have been a direct specific link to the issue.

PT09, patient interview

...when I looked at it, it was a lot of other things in there. I really had to hunt for anything that was directly related to me.

PT07, patient interview

#### Impact on Clinical Care

Due to the short study period, only 11 of the patients who took part in evaluation interviews had seen their treating doctor. These participants had mixed opinions about the value of discussing their PROMPT-Care assessment results during consultations. Only 3 patients recalled discussing their assessment report during consultations but felt that it facilitated communication and increased recognition and acknowledgment of their concerns:

Yes it did. It [PROMPT-Care] really did [help], because you were more than just the cancer. Your life is more than just cancer...And as a result, she [clinician] said did I want to be referred to a psychologist.

PT08, patient interview

It [PROMPT-Care] actually came up [in consultation] straight away and it actually gave me a handle to express something that I hadn’t—couldn’t figure out how to express to the person I was speaking too. And it prompted them to ask me questions.

PT01, patient interview

Additionally, another 3 patients indicated they would have liked to have discussed their responses with their specialist but were not given the opportunity. The remaining patients felt they had no need or did not see any benefit of discussions with their specialist, with one patient noting that the self-management resources adequately dealt with their concerns:

No, ’cause I think I’m quite an upbeat person and—yeah. I don’t think I would’ve gotten anything from that other than maybe what I’ve got from the website.

PT05, patient interview

Furthermore, the vast majority of interviewed patients indicated they saw great benefit in their general practitioner (GP) receiving a copy of their clinical feedback reports in the future. They felt that it would be a good approach for their GP to keep up to date on their cancer care. Patients also commented that it held potential to reduce unnecessary repetition of information and ensure that key information regarding their treatment and clinical care was not forgotten or overlooked. One patient noted:

Definitely. Definitely. I have no problems with them receiving anything and I think it saves me then having to go and then try to explain everything and I will forget things. It is the ideal for him to have as much information as they had about whatever is going on with me.

PT05, patient interview

**Table 2:** Summary of self-management resource evaluation (N=28).

Self-management resource evaluation		n (%)
**Overall satisfaction with resources^a^**		
	Satisfied or very satisfied	11 (39)
	Neutral	7 (25)
	Unsatisfied or very unsatisfied	2 (7)
**Time spent accessing resources, in minutes^a^**		
	0-10	9 (32)
	11-20	12 (43)
	21-30	0 (0)
	Greater than 30	1 (4)
**Place where resources were accessed^a^**		
	At home	17 (55)
	At work	2 (6)
	In a public place	2 (6)
	At a family or friend’s house	1 (3)
**Shared resources with others^a^**		
	Yes	5 (18)
	No	17 (61)
**Easy to access via email sent^a^**		
	Strongly agree or agree	17 (61)
	Neutral	4 (14)
	Strongly disagree or disagree	1 (4)
**Relevant to concerns^a^**		
	Strongly agree or agree	15 (54)
	Neutral	6 (21)
	Strongly disagree or disagree	1 (4)
**Easy to understand and navigate^a^**		
	Strongly agree or agree	17 (61)
	Neutral	4 (14)
	Strongly disagree or disagree	1 (4)
**Helped to personally deal with some concerns^a^**		
	Strongly agree or agree	7 (25)
	Neutral	8 (29)
	Strongly disagree or disagree	3 (11)

^a^Some level of missing data.

#### Oncology Staff Feedback

Overall, all staff reported high acceptability of the eHealth system. Oncology staff indicated that the PROMPT-Care system was a useful screening tool that allowed them to identify specific issues to raise with the patient during consultations, and the clinical feedback reports allowed them to adequately prepare for the upcoming consultation:

[PROMPT-Care] would sort of give a greater value to the time they spent because it would be troubleshooting in a very sort of quick way. So it’s a tool for troubleshooting. It gives better value for patients in terms of what they get out of the consultation.

HCP04, staff interview

Well, I quite liked the physical problems [checklist] because patients sometimes forget to tell us things, and/or they don’t think things are important...So, from that point of view, it sort of gave me a quick look and targets the consultation a bit more.

HCP01, staff interview

They also felt the clinical feedback reports enabled them to get to know their patients better, provided them with an in-depth understanding of their patients’ issues and needs, and created an opportunity to discuss sensitive topics:

I would have an impression about a patient that things weren’t going fantastically, but it [clinical feedback reports] gave greater granularity and specificity about where the needs were.

HCP04, staff interview

Oncology staff also suggested that PROMPT-Care brought patients back into the system when issues remained unresolved and enabled them to better support patients through referral to appropriate supportive care options and health care professionals:

A lady who I know has a very high level of anxiety. So, I contacted her because I was surprised that she had still identified these factors as I thought she had turned a corner. So, it was good from that point to catch up with her...So, I sent a letter to her GP notifying about that.

HCP02, staff interview

However, some oncology staff mentioned concern that some patient responses were not directly related to their cancer care and could lead to difficulty interpreting patient responses and problems. Additionally, some felt that certain emotional issues, such as anxiety and fear of cancer recurrence, could not be resolved regardless of information and support provided. They felt that these elements of care could possibly be better followed up by appropriate nursing teams who could either address these ongoing concerns or refer the patient onto the appropriate services:

Part [of the clinical feedback report] was sort of more about family problems that often have things in there that there were stressors with their partner or whatever. But when I’d explore that a bit further with them, it would actually have nothing to do with the oncology situation...But when you go back and take the history, it hasn’t been so good for the last 10 years. So, it’s picking up a lot of things that are not specifically related to malignancy.

HCP01, staff interview

Looking at a report, if there were flags beyond the biological component of the cancer...did make their consultations probably double in time. So, if that was followed up by the care coordinator or the CNC [clinical nurse consultant] along those lines, that they had the time for it.

HCP03, staff interview

Some oncology staff commented that the assessments collected a large and diverse amount of information and felt that they were unable to adequately review all items and address the issues in a single short clinical consult. They felt that this had the potential to increase clinical workloads and extend consultation times, raising new issues in service delivery and patient care:

It’s quite likely that the [PROMPT-Care] report has much deeper value or—significant value than my 3-minute or 5-minute time to explore these.

HCP04, staff interview

Additionally, one oncologist suggested that the assessments picked up issues that were already known to the clinical team, leading to information being communicated repetitively:

I...it’s just putting the nuance context of it on each patient because for some of these patients everything is red...and many of the areas were [already] being addressed, and so anybody coming to it [the consultation] cold wouldn’t really have that information and couldn’t really sort of graduate their questioning or directly questioning.

HCP05, staff interview

Throughout training and pilot testing, all staff demonstrated relatively high competency with technology and overall ability to use the OIS. However, all 5 staff reported difficulty identifying which patients were on the PROMPT-Care trial and also initially locating the clinical feedback reports within the OIS because of a lack of familiarity with navigating the sections of the OIS in which the PROMPT-Care reports were located. They felt that these issues needed to be resolved and included in ongoing staff training in how to use the hospital OIS generally to avoid decreased usage of the PROMPT-Care system over time.

## Discussion

The objectives of our feasibility study were to (1) develop a fully integrated eHealth system to support electronic assessment of cancer patients’ PROs, feedback of PRO results in real time to their treating clinicians, and support of patients’ self-management through generation of links to resources, which are tailored to their PROs; (2) implement the pilot version of PROMPT-Care at two hospitals and test the feasibility and functionality of the system and receive feedback to fine-tune any future system; and (3) test the acceptability of PROMPT-Care in a sample of cancer patients and clinicians at the two participating hospitals.

Overall, the results suggest that the PROMPT-Care eHealth system is both feasible and acceptable to the users, that is, the patients and cancer care team. This feasibility study also identified important modifications, particularly relating to patient assessment completion and clinician access to the reports, which should be undertaken to increase PROMPT-Care’s acceptability and feasibility before its large-scale implementation during the next trial phase and for future implementation as part of routine care.

### Patient Experience Completing Assessments

Data capture is a critical first step in any ePRO-based system. Study participants found the Web-based survey completion to be easy, consistent with published evidence of patients’ preference for this mode compared with paper versions of surveys [17]. The length of time taken to complete the assessments (mean: 15 min) was also highly acceptable. Importantly, the results indicate very low levels of missing data, which were predominantly the assessment start and completion times and some demographic variables. This may reflect a perception by patients that these items were not important for informing either their clinical care or self-management. In the context of implementing this system as routine care, these fields are in fact the least important, with assessment start or finish times only collected in this pilot study to determine assessment completion time, and patient demographic characteristics can be accessed by the care team via patients’ medical records through the OIS, when required. It is important to note that there were no other patterns of missing data observed.

The cognitive interviews highlighted areas where patients experienced some difficulties completing the PROMPT-Care assessment. First, the Distress Thermometer widget, which required patients to slide the cursor vertically to the number between 0 and 10 that best reflected their level of distress, was observed to be non-responsive for some patients or was not sliding to the exact number that patients were verbally reporting their distress score to be. As a result, modification for the next phase of this program will be made, with the thermometer widget abandoned in preference for a standard 11-point (0-10) scale item. Second, the differing time frames for each of the measures (ESAS *today*; Distress Thermometer and problem checklist *in the past week, including today*; and SCNS-ST9 *in the last month*) was unclear to some patients. As the PRO instruments used are validated, it is important to retain the time frames as per the originals. Therefore, the amendments for phase 2 will include provision of much clearer instructions that draw more attention to the time frame to consider when answering each item. Third, the cognitive interviews suggested that some of the patients’ responses were not specifically in the context of their cancer experience (eg, childcare and dealing with partner or family). This was confirmed by some oncology staff, who expressed concern that some patients’ responses were not directly related to their cancer care and could lead to difficulty interpreting patient responses and problems. Review of the PROMPT-Care assessment highlighted the need for greater specificity in the instructions for phase 2, which will ask patients to answer all questions only in relation to their cancer and cancer care experience. Finally, patient feedback suggested a need to simplify item response options to reduce response burden in the next phase of research (eg, only requiring patient to select *yes* if they were experiencing a particular Distress Thermometer Checklist issue—total of 39 issues—instead of having to select either *yes* or *no* for each of those issues).

### Informing Clinical Care

Integration of the PRO measures into the existing hospitals’ OISs was hypothesized to enhance their relevance and usefulness in informing routine cancer care [18,19]. However, it is worth noting that during this pilot phase, only 3 out of 11 patients who had appointments during the study period reported that their clinician discussed the PROMPT-Care report with them during their consultation. Whereas this suggests low clinician engagement, when the clinical feedback reports were reviewed, the clinicians felt that they enabled them to get to know their patients better, provided them with an in-depth understanding of their patients’ issues and needs, and created an opportunity to discuss sensitive topics. They also believed that the report facilitated them concentrating on the issues highlighted as important by the patients. Furthermore, the 3 patients who recalled discussing their assessment report during consultations felt that it facilitated communication and increased recognition and acknowledgment of their concerns. It is therefore perhaps not the usefulness of the reports that limited their use but potentially other reasons. Identifying the PROMPT-Care trial patients and accessing the clinical feedback reports proved to be problematic for the 5 clinicians involved in the pilot phase. Hence, the opportunity to review the PROMPT-Care report may have been missed for the remaining 8 patients who had a consultation during the pilot phase. The clinicians interviewed in this study expressed the need for these issues to be resolved and included the importance of ongoing staff training, particularly with regard to accessing the reports within the OIS once assessment data had been imported. They also indicated that others from the cancer care team, such as the nurses, are well placed to review the PROMPT-Care reports and to act on their recommendations. It is also important to note that patients also saw great benefit in their GPs receiving reports detailing their treatment and clinical care, as a useful tool to enhance communication and reduce information repetition. These results underscore important modifications for the next phase of research, in particular, streamlining accessibility to the PROMPT-Care report and training all members of the cancer care team to access and respond to report content.

In the pilot configuration of the PROMPT-Care system (version 1), clinicians received an alert via the OIS when a PROMPT-Care patient was attending an appointment, which served as a trigger to review their report. However, the pilot study highlighted a need for alerting the cancer care team when PROMPT-Care patients who did not have a scheduled appointment (eg, follow-up patients) reported unresolved issues. As a result, version 2 of the PROMPT-Care system will incorporate a clinical email alert to inform the cancer care team of patients with ongoing issues to trigger appropriate action as per that cancer center’s care agreed pathway.

### Supporting Patient Self-Management

In addition to informing clinical care, the other key feature of the PROMPT-Care program was to support patient self-management, with patients receiving links to pages of information and resources based on their PROs. Patient feedback suggests that they valued the resources, appreciated having access to reliable information, and felt that the information prompted them to be more engaged in their own health. However, in version 1 of PROMPT-Care, patients received the domain page relevant to their concern even if they only reported one item above threshold. For example, if a patient only reported above-threshold pain, she or he would be sent the physical well-being page, which contains access to 29 resources. This resulted in only approximately half of the participants reporting the self-management resources to be directly relevant to their concerns. These results highlight the need for review and improvement of the self-management resources and consideration of a tiered approach in PROMPT-Care version 2, with generic information resources available for patients initially reporting an issue above threshold but more dynamic and interactive resources (eg, videos, podcasts, or interactive self-help programs such as *Coping-Together* [20] or the Cancer Council ENRICH [Exercise and Nutrition Routine Improving  *Cancer* Health] survivorship program [21]) available when issues remain unresolved on a subsequent PROMPT-Care assessment.

### Conclusions

An issue that remains unresolved from this pilot is the recommended frequency of PROMPT-Care assessments, with a diversity of responses from patients regarding this issue. This suggests that further evaluation of this aspect is required in the next phase of our research, particularly exploring whether different assessment frequency is required for patients on treatment versus in follow-up.

To our knowledge, this is the first study piloting an integrated PRO eHealth system in the Australian health care context. Although the patient participant numbers were small, our sample aligns with feasibility and usability testing recommendations, which suggest that a sample of 30 to 40 will allow for 97% to 98% of usability problems to be identified [22]. Additionally, Nielsen and Landauer’s model suggests that most usability problems can be detected by 10 users and 50% by 5 users [23]. Therefore, although some of our evaluation numbers are low, they are consistent with other similar feasibility and acceptability studies [24-27] and sufficient to evaluate the acceptability and feasibility of version 1 of PROMPT-Care and to identify modifications required for version 2 to utilize in the next stage of large-scale testing.

The nature of this study also meant that we neither evaluate the frequency of patients’ accessing of self-management resources nor the oncology staff’s use of clinical feedback reports. These utility elements of the PROMPT-Care system will be evaluated within a larger future study.

## Abbreviations

CINSW: Cancer Institute NSW
eHealth: electronic health
ePRO: electronic patient-reported outcome
ESAS: Edmonton Symptom Assessment Scale
GP: general practitioner
OIS: oncology information system
PRO: patient-reported outcome
PROMPT-Care: Patient-Reported Outcome Measures for Personalized Treatment and Care
SCNS-ST9: Supportive Care Needs Survey–Screening Tool 9
